# Effect of β-Caryophyllene on PPAR-γ, NF-κB, and CNR2: Implications for Gut–Brain Axis Communication in a Murine Model of Diet-Induced Obesity

**DOI:** 10.3390/metabo15100638

**Published:** 2025-09-24

**Authors:** Cristina Pech-Jiménez, Lucrecia Carrera-Quintanar, Juan Manuel Viveros-Paredes, Yolanda Fabiola Marquez-Sandoval, Luis Felipe Jave-Suárez, Adelaida Sara Minia Zepeda-Morales, Gilberto Velázquez-Juárez, Rocio Ivette López-Roa

**Affiliations:** 1 Doctorado en Ciencias de la Nutrición Traslacional, Departamento de Alimentación y Nutrición, Centro Universitario de Ciencias de la Salud, Universidad de Guadalajara, Jalisco 44350, Mexico; elma.pech3708@alumnos.udg.mx (C.P.-J.);; 2 Laboratorio de Investigación y Desarrollo Farmacéutico, Departamento de Farmacología, Centro Universitario de Ciencias Exactas e Ingenierías, Universidad de Guadalajara, Jalisco 44430, Mexico; 3 Instituto de Investigación en Cáncer en la Infancia y la Adolescencia, Departamento de Reproducción Humana, Crecimiento y Desarrollo Infantil, Centro Universitario de Ciencias de la Salud, Universidad de Guadalajara, Jalisco 44350, Mexico; 4 División de Inmunología, Centro de Investigaciones Biomédicas de Occidente (CIBO), Instituto Mexicano del Seguro Social, Jalisco 44340, Mexico; lfjave@gmail.com; 5 Laboratorio de Análisis Clinicos e Investigación Traslacional, Departamento de Farmacobiología, Centro Universitario de Ciencias Exactas e Ingenierías, Universidad de Guadalajara, Guadalajara 44430, Mexico; 6 Departamento de Química, Centro Universitario de Ciencias Exactas e Ingenierías, Universidad de Guadalajara, Guadalajara 44430, Mexico

**Keywords:** β-caryophyllene, obesity, inflammatory, mice

## Abstract

Background /Objectives: The rising prevalence of metabolic disorders, such as obesity, is linked to increased consumption of high-calorie foods and sedentary lifestyles. While conventional treatments rely on lifestyle modifications and pharmaceuticals, these often have limitations and adverse effects. As an alternative, natural compounds like β-caryophyllene (BCP), found in spices such as black pepper and cloves, have gained interest due to their anti-inflammatory and metabolic properties. This study investigated the effects of BCP on the gut–brain axis in obese C57BL/6J mice. Methods: Quantitative real-time PCR (RT-qPCR) was performed using a Rotor-GeneQ thermocycler (Qiagen). Relative gene expression levels were normalized to the reference gene’s transcript levels (2^−∆∆Ct^ method). Results: BCP was found to modulate key receptors, including FFAR3, LEPR, and GHSR, which are involved in appetite regulation and insulin sensitivity. Its action on the CNR2 (CB2 receptor) suggests additional benefits in energy balance and anorexigenic activity. Conclusions: These findings support BCP’s potential as a complementary therapy for obesity, though further studies are needed to confirm its efficacy in humans. Its safety profile and multifactorial effects make it a promising alternative to conventional treatments.

## 1. Introduction

The prevalence of metabolic disorders is on the rise, largely due to the increased availability of high-calorie foods and a decline in physical activity [1]. According to a report by the World Health Organization (WHO) published in June 2021, the global prevalence of obesity has nearly tripled since 1975. In 2016, it was reported that more than 1.9 billion adults worldwide were overweight, of whom over 650 million were obese [2]. Current obesity therapies primarily rely on lifestyle modifications, including reduced caloric intake and increased physical activity, complemented by pharmacological approaches that suppress appetite, enhance satiety, or limit fat absorption [3].

However, these strategies are not always sufficient or sustainable in the long term, and synthetic pharmacological agents, such as liraglutide, phentermine, and naltrexone, among others [4,5], have been associated with significant adverse effects. In this context, bioactive compounds derived from sustainably cultivated plants have emerged as a promising alternative, offering not only an eco-friendly approach but also additional health benefits beyond weight control [6,7,8].

These naturally derived compounds exhibit a more favorable safety profile compared to synthetic pharmaceuticals, making them an attractive option for obesity management. Furthermore, their mechanism of action is characterized by targeting multiple molecular pathways and physiological processes, including the regulation of metabolic functions, modulation of gene expression related to lipid and carbohydrate metabolism, and influence on gut microbiota composition and functionality. This versatility positions them as valuable tools in the development of comprehensive and personalized therapies to combat obesity and its associated comorbidities [9,10,11].

Molecules derived from a plant origin demonstrate promising anti-obesity therapeutic potential by improving metabolic parameters and reducing inflammation. Critically, the majority of these compounds, including berberine blends [12,13], epigallocatechin-3-gallate [14,15,16], and various botanical extracts [17,18,19,20], reported comprehensive safety profiles with no significant adverse effects. While some studies noted transient, minor alterations in liver enzymes (AST, ALT) or gastrointestinal discomfort, these were not deemed severe. The consistent reporting of main and adverse effects across most randomized controlled trials indicates a high level of safety monitoring and suggests that these plant-derived molecules are generally well-tolerated in obesity management.

β-caryophyllene (BCP), a natural bicyclic sesquiterpene found in various plant species, including those of the Cannabis genus and other aromatic species such as cloves, black pepper, and oregano, is one of the most studied and promising natural compounds [21]. Its therapeutic potential is primarily attributed to its well-characterized role as a selective cannabinoid receptor type 2 (CB2) agonist, a mechanism that underpins its potent anti-inflammatory and immunomodulatory properties. Notably, emerging evidence indicates that BCP also confers significant beneficial effects on critical metabolic parameters. These include the amelioration of insulin resistance, the reduction in hepatic steatosis, and the modulation of lipid metabolism. Importantly, BCP has been evaluated in human clinical studies, which have confirmed its safety and good tolerability profile at effective doses. Reported adverse effects are typically mild and infrequent (e.g., occasional nausea or headache), with no serious adverse events documented [22].

Building on our earlier finding that BCP improves intestinal integrity in mice [23], we previously evaluated its broader immunomodulatory effects on serum endotoxemia levels, short-chain fatty acid (SCFA) concentration, the abundance of bacterial phyla in the colon, and serum leptin levels. Given the growing evidence suggesting that natural compounds can modulate signaling pathways involved in obesity and inflammation, this study aims to evaluate the effects of BCP on markers belonging to the gut–brain axis in obese mice.

## 2. Materials and Methods

This study utilized immunofluorescence techniques to detect transcription factors and gene expression analysis to investigate the mechanisms of communication within the gut–brain axis. As previously described in Rodríguez-Mejía et al. (2022) [23] , the in vivo experimental design employed C57BL/6J mice divided into four groups: a control group fed a standard diet (STD, *n* = 6), a standard diet supplemented with BCP (STD + BCP, *n* = 6), a high-fat diet group (HFD, *n* = 6), and a high-fat diet plus BCP group (HFD + BCP, *n* = 6). Following one week of acclimatization under standard dietary conditions and ad libitum water access, Mice in the BCP groups were administered a molecule at a dosage of 50 mg/kg body weight daily. The compound was dissolved in a vehicle of 4% saline with Tween 80 (Sigma Aldrich, Sofia, Bulgaria, #P4780) and administered via oral gavage for the full 16-week (112-day) experimental period. The minimum sample size for Western blot and RT-qPCR was calculated to ensure 80% statistical power for confirming the difference in interest, based on the finite number of samples and the target transcription factors, according to Youssef et al. (2019) [24]. This design enabled a comparative evaluation of BCP’s effects under both normal conditions and diet-induced obesity (see Appendix A.1.)

We examined the transcription factors PPAR-γ (peroxisome proliferator-activated receptor gamma) and NF-κB (nuclear factor kappa-B) in intestinal tissue. Simultaneously, we analyzed the expression of key markers in brain tissue, including FFAR3 (free fatty acid receptor 3), which senses microbial metabolites; the leptin (LEPR) and ghrelin (GHSR) receptors, involved in appetite regulation; and the endocannabinoid receptors CNR1 and CNR2, which modulate energy balance.

### 2.1. Western Blot

For protein quantification, samples from the duodenum, jejunum, ileum, and colon were homogenized in RIPA lysis buffer (Thermo Fisher Scientific, Waltham, MA, USA, #89901) containing a protease and phosphatase inhibitor cocktail (Thermo Fisher Scientific, #A32961) using a TissueLyser II (Qiagen, Hilden, Germany). The homogenates were centrifuged at 14,000 rpm for 15 min at 4 °C, and the supernatants were collected. Protein concentrations were determined using a Bicinchoninic acid (BCA) assay (Thermo Fisher Scientific, #23235) following the manufacturer’s instructions.

For quantitative Western blot analysis, samples were denatured in 6 × Laemmli buffer at 95 °C, and 10 µg of protein was loaded onto 10% SDS-PAGE gels. Proteins were transferred to polyvinylidene fluoride (PVDF) membranes using a Trans-Blot^®^ SD Semi-Dry Transfer Cell (Bio-Rad, Hercules, CA, USA, #1703940). The membranes were blocked with Odyssey Blocking Buffer (Li-Cor, Lincoln, NE, USA) for two hours and then incubated with primary antibodies: mouse polyclonal anti-PPAR-γ (1:1000, Cell Signaling Technology, Danvers, MA, USA, #95128) and anti-phospho-NF-κB p65 (1:1000, Cell Signaling Technology, #3036). Subsequently, the membranes were incubated with IRDye^®^ 800CW donkey anti-mouse IgG (1:15,000, Li-Cor, #926-32212) for 90 min at room temperature, followed by PBS washes. Protein bands were visualized and quantified using an Odyssey^®^ CLx Imaging System (Li-Cor Biosciences) at 800 nm. Optical density (OD) was measured using Image Studio Lite software (v5.2.5) and normalized to Revert™ 700 Total Protein Stain (Li-Cor, #11010) for densitometric analysis.

### 2.2. Reverse Transcription—(RTq)—PCR

Total RNA was isolated from hypothalamic tissue using TRIzol reagent (Life Technologies, #15596018) via phenol-chloroform extraction, followed by spectrophotometric quantification (NanoDrop™, Thermo Scientific). cDNA was synthesized from purified RNA using the iScript cDNA Synthesis Kit (Bio-Rad, #1708841). Gene expression analysis was performed using SYBR Green (Bio-Rad, #172-5270) in quantitative PCR (qPCR) assays on a Rotor-Gene Q cycler (Qiagen). Each reaction contained 30 pg of cDNA template, 5 µL of SYBR Green/ROX qPCR Master Mix 2 × (Bio-Rad, #1725150), 10 µM of forward and reverse primers (see Appendix A.2), and nuclease-free water to a final volume of 10 µL. GAPDH was used as the endogenous control. The amplification protocol consisted of an initial denaturation step at 95 °C for 30 s, followed by 35 cycles of denaturation at 95 °C for 5 s and annealing at 60 °C for 34 s. A final incubation at 50 °C for 30 s and 20 °C for 10 s was included. Relative gene expression levels were calculated using the 2^−ΔΔCt^ method and normalized to GAPDH.

### 2.3. Statistical Analysis

Statistical analyses were performed using GraphPad Prism software (version 8.1, San Diego, CA, USA). Data are presented as mean ± standard deviation (SD). Significant differences between groups (* *p* < 0.05; ** *p* < 0.01; *** *p* < 0.001; **** *p* < 0.0001). were determined using the Kruskal–Wallis test, followed by Dunn’s U post hoc test to identify specific intergroup differences under the experimental conditions.

## 3. Results

### 3.1. Differential Regulation of PPAR-γ and NF-κB/p65 by BCP in the Small Intestine and Colon

**Figure 1:**
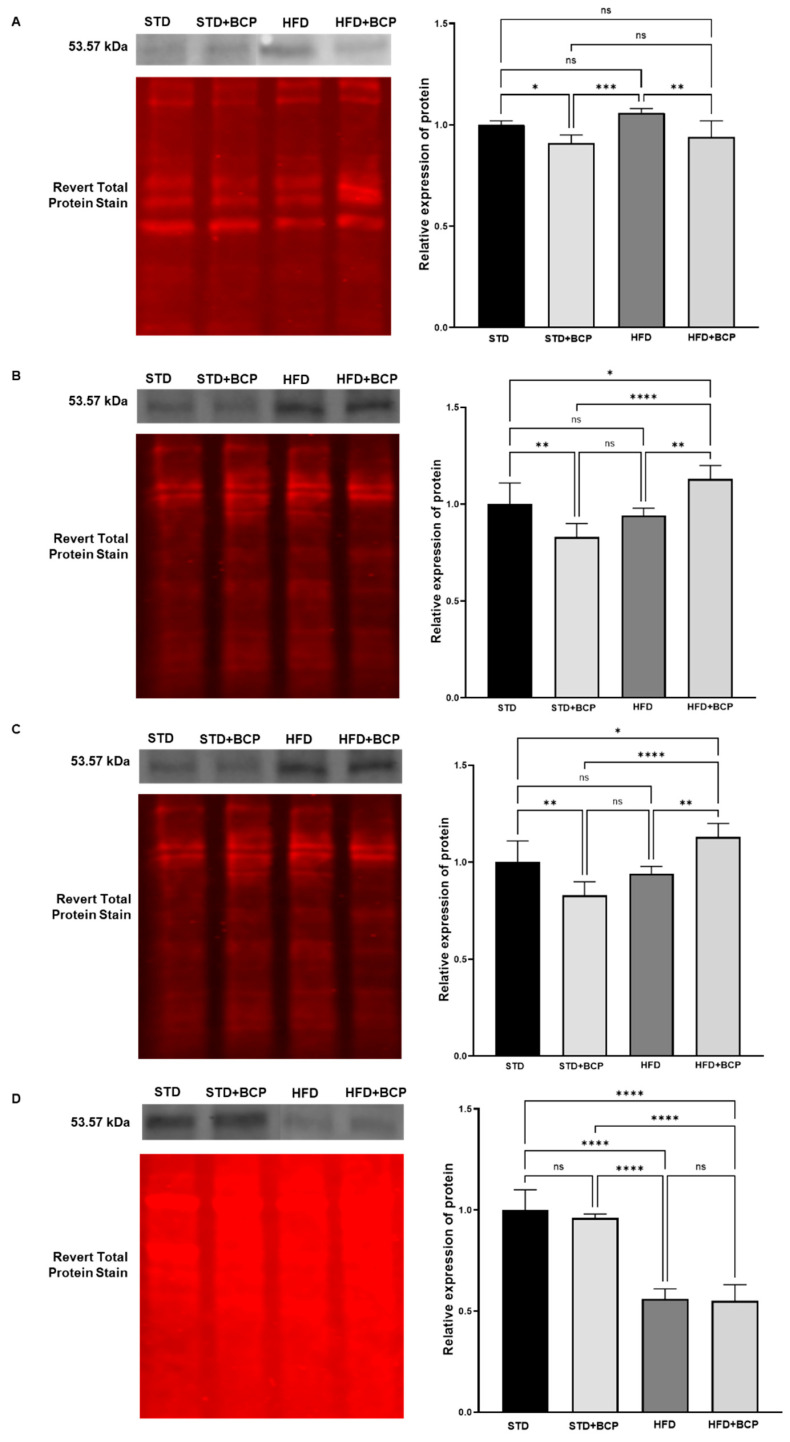
Effects of BCP on PPAR-γ in (**A**) duodenum, (**B**) jejunum, (**C**) ileum, and (**D**) colon tissues under different dietary conditions. (STD) Standard diet; (STD + BCP) Standard diet + BCP; (HFD) High-fat diet; (HFD + BCP) High-fat diet + BCP. Data are presented as mean signal intensity (*n* = 6) ± SD, derived from densitometric analysis. Statistical differences were assessed using the Kruskal–Wallis test followed by Dunn’s post hoc tests (* *p* < 0.05; ** *p* < 0.01; *** *p* < 0.001; **** *p* < 0.0001; ns, not significant).

This section presents the results evaluating the effects of BCP on the transcription factor PPAR-γ in the small intestine and colon. PPAR-γ is a key transcription factor regulating metabolic and anti-inflammatory processes in various tissues, including the gastrointestinal system [25, 26] . Figure 1 shows the effects of BCP on the PPAR-γ signaling pathway in small intestinal tissue under different dietary conditions. Western blot and fluorescence imaging revealed differential PPAR-γ expression under the four aforementioned experimental conditions. The band patterns and fluorescent signals demonstrate PPAR-γ modulation, showing changes associated with both diet type and BCP treatment.

In duodenal tissue samples (Figure 1A), the effects of BCP on the activation of the PPAR-γ signaling pathway are clearly visualized in the left panel of the figure. In duodenal tissue (Figure 1A), the analysis revealed a significant decrease in PPAR-γ activation in the group fed a high-fat diet supplemented with BCP (HFD + BCP), compared to the HFD group (*p* < 0.01). This result suggests that, under conditions of metabolic stress induced by a high-fat diet, BCP exerts a negative modulatory effect on PPAR-γ signaling, which could imply an unfavorable impact on processes regulated by this transcription factor.

In jejunal tissue analyses (Figure 1B), PPAR-γ expression revealed that the STD + BCP group exhibited a downregulation compared to the STD group (*p* < 0.001). In contrast, in the HFD group, an increase in PPAR-γ signal was observed relative to STD + BCP. However, this effect was more pronounced in the HFD + BCP group compared to HFD alone (*p* < 0.01). Statistical analysis confirmed significant differences between groups, particularly between STD + BCP and HFD + BCP (*p* < 0.0001). Significant differences were also observed between the STD and HFD + BCP, with the latter showing the highest expression levels of PPAR-γ protein (*p* < 0.05).

Figure 1C illustrates the effects of BCP on PPAR-γ synthesis in ileal tissue under different dietary conditions. In the STD group, the intensity of the PPAR-γ signal was higher compared to STD + BCP, and this difference was statistically significant (*p* < 0.01). When comparing STD with HFD + BCP, the PPAR-γ signal was significantly higher in the latter group (*p* < 0.05). Similarly, when comparing STD + BCP with HFD + BCP, the latter group showed a significantly greater PPAR-γ signal (*p* < 0.0001). Finally, in the comparison between HFD and HFD + BCP, a higher protein level was also observed in the latter group (*p* < 0.01).

The results from colonic tissue (Figure 1D) indicate that the STD group displayed higher PPAR-γ synthesis compared to STD + BCP, where a slight decrease in PPAR-γ signal was observed, although this difference was not statistically significant. The comparison between STD and HFD showed a statistically significant reduction in PPAR-γ signal (*p* < 0.0001), indicating that obesity downregulates the activation of this pathway in the colon. Significant differences were observed between the STD and HFD + BCP groups (*p* < 0.0001). Meanwhile, the comparison between STD + BCP and HFD revealed a downregulation of PPAR-γ signaling (*p* < 0.0001). Furthermore, when comparing STD + BCP and HFD + BCP, the latter group exhibited a further downregulation of this protein (*p* < 0.0001).

**Figure 2:**
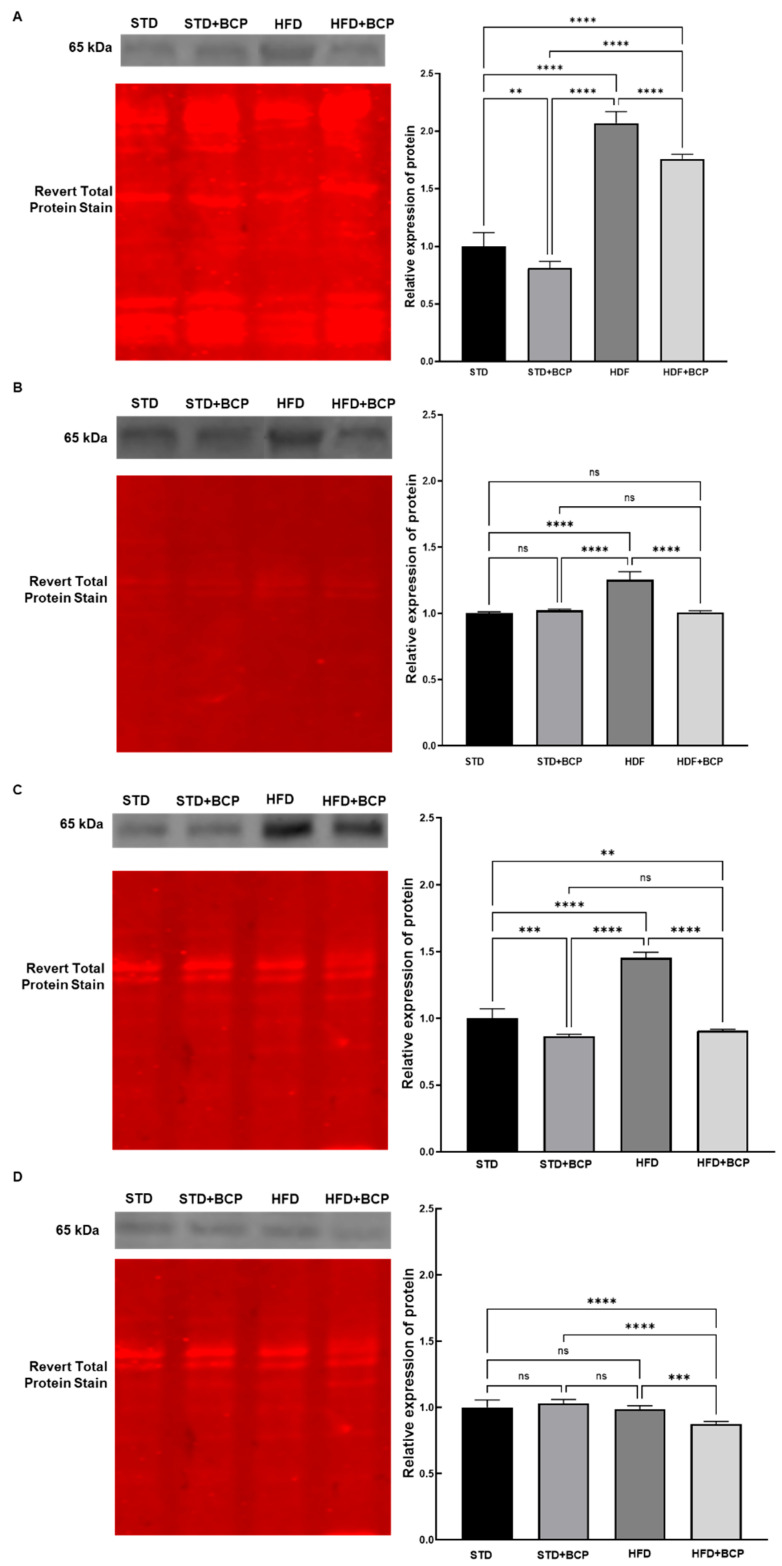
Effects of BCP on NF-κB/p65 in (**A**) duodenum, (**B**) jejunum, (**C**) ileum, and (**D**) colon tissues under different dietary conditions. (STD) Standard diet; (STD + BCP) Standard diet + BCP; (HFD) High-fat diet; (HFD + BCP) High-fat diet + BCP. Data are presented as mean signal intensity (*n* = 6) ± SD, derived from densitometric analysis. Statistical differences were assessed using the Kruskal–Wallis test followed by Dunn’s post hoc tests (* *p* < 0.05; ** *p* < 0.01; *** *p* < 0.001; **** *p* < 0.0001; ns, not significant).

The transcription factor NF-κB/p65 is a key regulator of inflammatory response and cellular stress in gastrointestinal tissues [25]. The expression levels of NF-κB/p65 in the duodenum under different dietary conditions are presented in Figure 2A. The STD group showed the highest signal intensity, which was significantly reduced in the STD + BCP group (*p* < 0.01). Conversely, the expression under the STD was downregulated compared to the HFD and HFD + BCP groups (*p* < 0.0001). The STD + BCP group exhibited lower NF-κB/p65 expression than both the HFD and HFD + BCP groups (*p* < 0.0001). A notable activation was observed in the HFD group compared to the STD, STD + BCP, and HFD + BCP groups. Statistical significance between groups confirms that the impact of BCP is dependent on the dietary context (*p* < 0.0001).

In the jejunum samples (Figure 2B), statistical analysis revealed a significant difference in NF-κB/p65 expression. The STD group showed lower expression compared to the HFD group (*p* < 0.0001). Similarly, the STD + BCP group exhibited reduced expression relative to the HFD group. Furthermore, a comparison between the HFD and HFD + BCP groups showed that NF-κB/p65 activation was decreased in the HFD + BCP group (*p* < 0.0001).

Figure 2C illustrates the response of the ileum to BCP under different dietary conditions. NF-κB/p65 expression was statistically significantly higher in the STD group compared to the STD + BCP (*p* < 0.001) and HFD + BCP groups (*p* < 0.0001). In contrast, the HFD group displayed a markedly increased NF-κB/p65 signal compared to the STD, STD + BCP, and HFD + BCP groups (*p* < 0.0001).

The measurement of NF-κB/p65 in the colon revealed a distinct pattern (Figure 2D). The NF-κB/p65 signal in the HFD + BCP group was statistically lower than that in the STD (*p* < 0.0001), STD + BCP (*p* < 0.0001), and HFD groups (*p* < 0.001).

### 3.2. BCP Modulates the Gene Expression of Hypothalamic Receptors for SCFAs, Leptin, and Ghrelin

**Figure 3:**
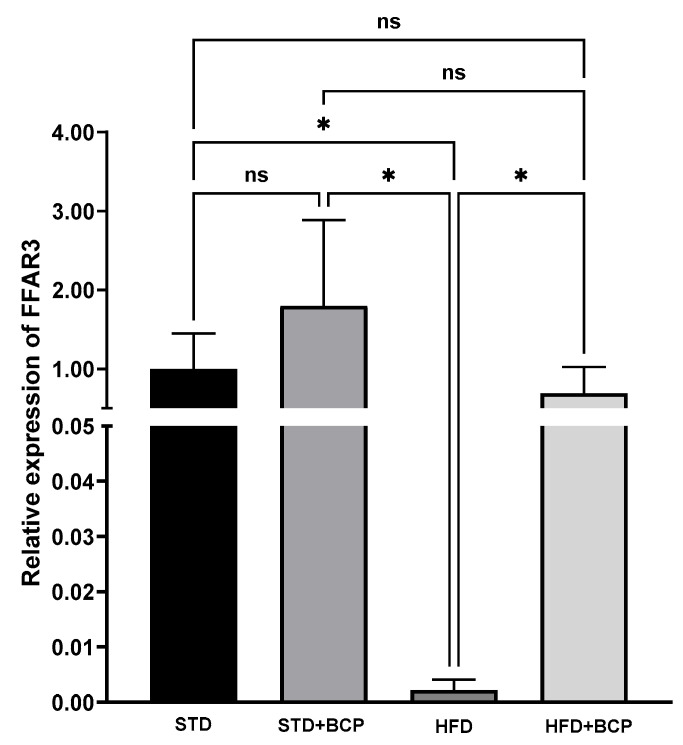
Effect of BCP on hypothalamic SCFA receptor (FFAR3) gene expression under different dietary conditions. (STD) Standard diet; (STD + BCP) Standard diet + BCP; (HFD) High-fat diet; (HFD + BCP) High-fat diet + BCP. Data represent mean values (*n* = 6) ± SD. Statistical differences were assessed by Kruskal–Wallis test followed by Dunn’s post hoc tests (* *p* < 0.05; ** *p* < 0.01; *** *p* < 0.001; **** *p* < 0.0001; ns, not significant).

As part of this investigation, we evaluated the impact of BCP on the gene expression of FFAR3, a receptor that plays a crucial role in modulating hunger and satiety processes by acting as a sensor for microbiota-derived metabolites [27] . Figure 3 illustrates the effects of BCP on FFAR3 receptor gene expression in the hypothalamus of a murine model of diet-induced obesity. Our analysis revealed no significant differences in FFAR3 gene expression between the standard diet (STD) and BCP-supplemented standard diet (STD + BCP) groups. However, comparison between the STD and high-fat diet (HFD) groups showed a significant difference (*p* < 0.05), indicating that the hypercaloric diet significantly downregulated FFAR3 gene expression compared to the standard diet. This difference was also evident when comparing the STD + BCP and HFD groups, where a significant variation was observed (*p* < 0.05). These results demonstrate that BCP exerts an upregulatory effect on FFAR3 expression.

A particularly significant observation emerged from the comparison between HFD and HFD + BCP groups, which revealed a statistically significant difference in FFAR3 gene expression (*p* < 0.05). This finding demonstrates that BCP exerts a substantial modulatory effect on FFAR3 expression, suggesting its potential to partially counteract the downregulatory impact of a high-fat diet on this receptor. These results indicate that BCP functions as an effective modulator of hypothalamic FFAR3 gene expression, particularly when the nutraceutical is present in the dietary regimen. The data support the hypothesis that BCP may help mitigate diet-induced impairment of this crucial metabolic signaling pathway.

**Figure 4:**
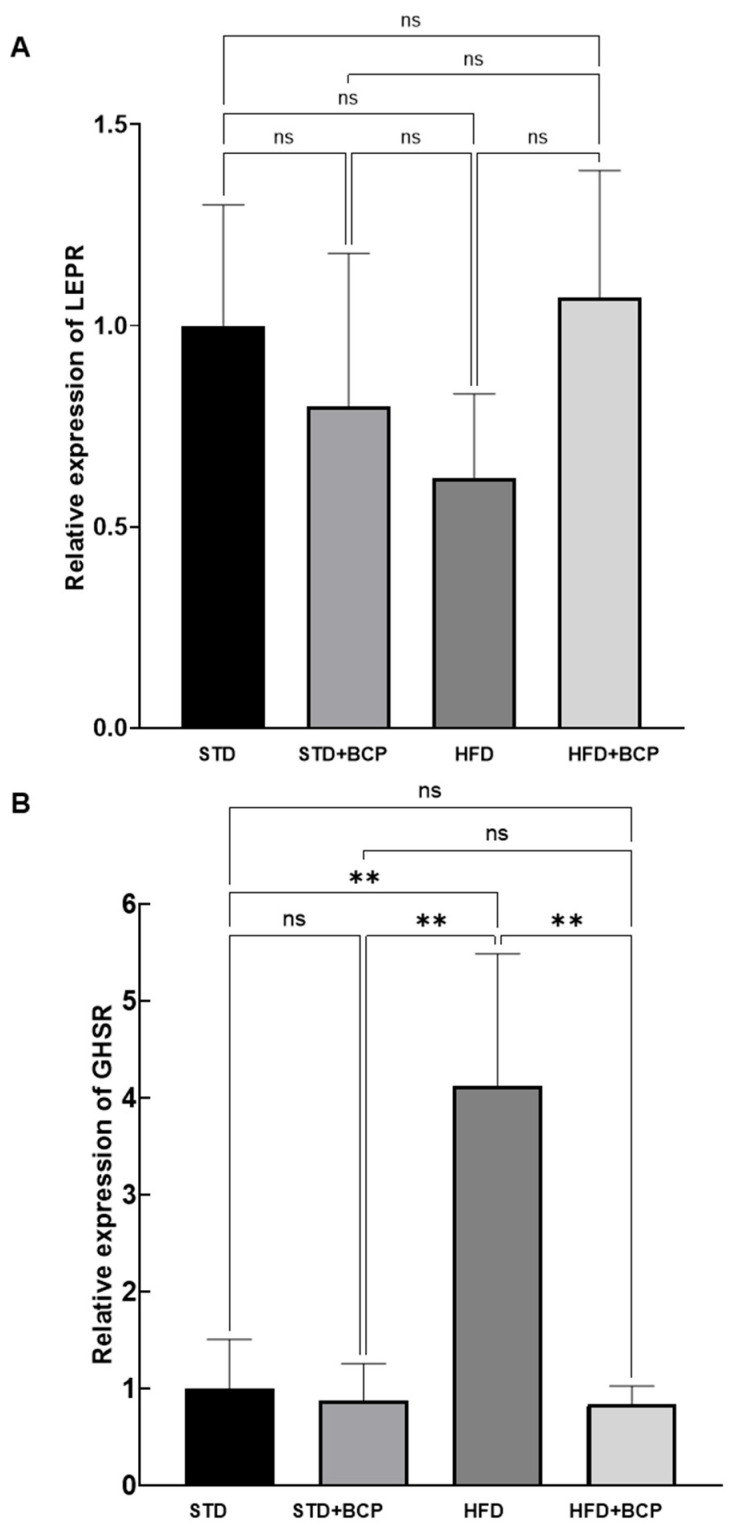
Effect of BCP on hypothalamic leptin receptor (LEPR) (**A**) and ghrelin receptor (GHSR) (**B**) gene expression under different dietary conditions. (STD) Standard diet; (STD + BCP) Standard diet + BCP; (HFD) High-fat diet; (HFD + BCP) High-fat diet + BCP. Data are presented as mean ± SD (*n* = 6). Statistical differences were assessed by Kruskal–Wallis test followed by Dunn’s post hoc tests (* *p* < 0.05; ** *p* < 0.01; *** *p* < 0.001; **** *p* < 0.0001; ns, not significant).

We also examined the gene expression of LEPR (leptin receptor) and GHSR (ghrelin receptor) in the hypothalamus to assess their regulation in response to BCP treatment. As shown in Figure 4, LEPR expression did not exhibit statistically significant differences among the experimental groups. However, we observed a modest upward trend in LEPR expression in the HFD + BCP group compared to the HFD group, suggesting a potential modulatory effect of the nutraceutical BCP under hypercaloric diet conditions. Although this difference did not reach statistical significance, the trend may indicate that BCP supplementation contributes to leptin receptor regulation in the context of diet-induced obesity.

The results for GHSR expression (Figure 4) revealed that the HFD group exhibited the most pronounced increase in receptor expression compared to all other groups, confirming that the hypercaloric diet model significantly upregulates GHSR gene expression. In contrast, the STD, STD + BCP, and HFD + BCP groups showed substantially lower expression levels, with similar trends among them. This pattern suggests that under standard diet conditions (with or without BCP supplementation) GHSR expression remains at lower levels than those observed in obesity conditions. Notably, BCP supplementation in the HFD + BCP group appeared to attenuate the marked increase observed in the HFD group, indicating a potential modulatory effect of the nutraceutical (*p* < 0.01). This finding is particularly relevant as it suggests that BCP may partially counteract the negative effects of the hypercaloric diet on GHSR expression. The data support that BCP exerts a downregulatory effect on GHSR expression in the context of a hypercaloric dietary regimen.

### 3.3. Effect of BCP on Hypothalamic Endocannabinoid System Receptor Expression

**Figure 5:**
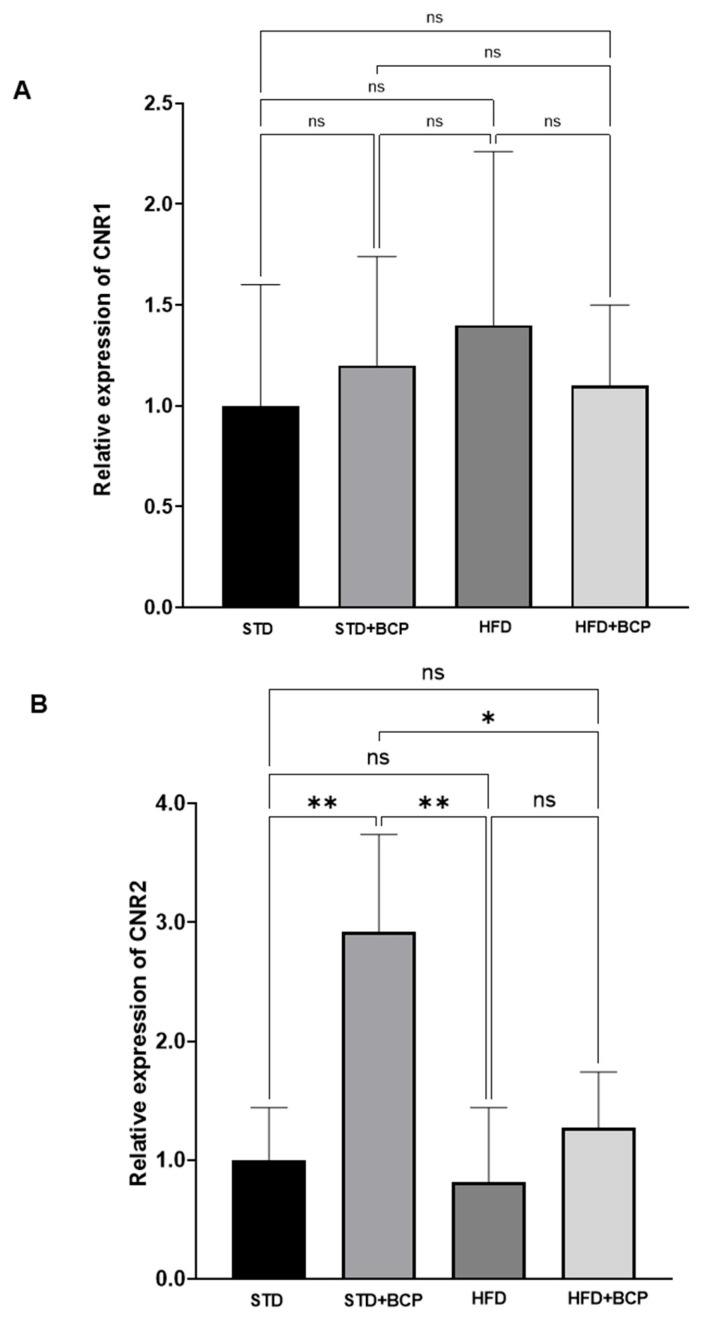
Effect of BCP on hypothalamic endocannabinoid system receptor gene expression CNR1 (**A**) and CNR2 (**B**) under different dietary conditions. (STD) Standard diet; (STD + BCP) Standard diet + BCP; (HFD) High-fat diet; (HFD + BCP) High-fat diet + BCP. Data are expressed as mean ± SD (*n* = 6). Statistical differences were determined by Kruskal–Wallis test followed by Dunn’s post hoc tests (* *p* < 0.05; ** *p* < 0.01; *** *p* < 0.001; **** *p* < 0.0001 ; ns, not significant).

The effect of BCP on the gene expression of ECS receptors in the hypothalamus was determined, evaluating its potential modulation under various experimental conditions. Figure 5 analyzes the behavior of the CNR1 gene expression in the hypothalamus of the murine model. Although no statistically significant differences were detected between the groups, some interesting trends were observed. In particular, the group fed the HFD showed a slight increase in CNR1 expression compared to the group that maintained the STD. On the other hand, the groups that received BCP supplementation (STD + BCP and HFD + BCP) showed expression levels similar to those of the control group, with a slight tendency to be lower than those of the HFD group. Although the multiple comparisons did not yield significant results, the data distribution suggests that CNR1 expression could vary depending on the type of diet and the presence of nutraceuticals such as BCP.

When evaluating the gene expression of the CNR2 receptor in brain tissue (Figure 5B), significant differences were observed between the STD group and the STD + BCP group (*p* < 0.01). On the contrary, no significant differences were detected between the STD group and the group subjected to an HDF and HDF + BCP diet, in which a reduction in CNR2 expression was recorded. However, when comparing the STD group with the HDF + BCP group, a trend of increasing CNR2 expression was observed, although this trend did not reach statistical significance.

A significant difference was detected between the STD + BCP and HDF groups, where the latter showed a lower expression of CNR2 (*p* < 0.01). On the other hand, when comparing the STD + BCP and HDF + BCP groups, a significant difference was observed, indicating that the STD + BCP group has a higher expression of this receptor compared to the HFD + BCP (*p* < 0.05). This finding highlights the potential role of BCP supplementation in obese conditions. Taken together, these results suggest that BCP supplementation induces a significant increase in CNR2 expression in the group exposed to the hypercaloric diet compared to the STD group and the HFD group. These findings suggest a modulating effect of BCP on the expression of the CNR2 receptor in obese conditions, which could have significant implications in the regulation of the endocannabinoid system in altered metabolic contexts.

## 4. Discussion

The bidirectional regulation between the gut and the brain is a complex mechanism affected by socioeconomic, genetic, environmental, dietary, and pharmacological factors [28]. This communication involves both neuromotor, immunological, and metabolic processes, so that an imbalance in one of these elements can have a significant impact on the others. In addition to the enteric neural network, the gut microbiota plays a crucial role in producing metabolites, such as SCFAs and secondary bile acids, which stimulate the secretion of intestinal peptides [29]. In this study, we evaluated how BCP affects the small intestine and colon through the synthesis of inflammation-related transcription factors PPAR-γ and NF-κB/p65), observing significant changes in the expression of these markers.

The nuclear receptor PPAR-γ is a key regulator of inflammatory processes, being widely studied in the context of obesity [30]. Its ligands exert anti-inflammatory effects by decreasing the production of proinflammatory cytokines (IL-8, TNF-α) and the expression of adhesion molecules [31,32]. Various studies have indicated that the action of BCP can be mediated in part by the activation of PPAR-γ through CNR2 receptor signaling, reinforcing its therapeutic potential in obesity [21,33].

In our work, the results on the expression of PPAR-γ in the small intestine showed unexpected behavior; the STD supplemented with BCP showed a decrease, while in the group with the HFD, a significant increase in PPAR-γ in the duodenum was observed. This finding is contrary to the literature, because PPAR-γ in the obesity process is modulated downwards [24,26,34]. In particular, the nutraceutical administered in the HFD showed increased synthesis in jejunum and ileum, having an expected effect in these sections.

In contrast to what was observed in the small intestine, the hypercaloric diet induced a significant reduction in PPAR-γ synthesis in the colon, an effect that the administration of BCP did not reverse. This finding is particularly unpredictable, given the known role of PPAR-γ as a transcription factor with protective effects against inflammatory and metabolic processes [24,35,36]. However, the increase observed in the HFD group could be interpreted as an initial compensatory mechanism of the organism against the metabolic stress induced by the high-fat diet. The increase in PPAR-γ expression in the duodenum could represent an attempt to maintain lipid homeostasis and insulin sensitivity in the early stages of metabolic dysfunction.

The decrease in PPAR-γ in the colon could be interpreted as a physiological response aimed at prioritizing the protection of other critical organs against metabolic stress derived from adiposity. This dual effect of PPAR-γ activation suggests that its downregulation in a specific tissue could mitigate systemic damage, even at the expense of less local protection. Thus, behavior in the colon could represent a metabolic balance to preserve the function of more vulnerable organs during obesity [30,37,38].

Chronic activation of the NF-κB/p65 pathway constitutes a central axis in obesity-associated inflammation, promoting the sustained release of proinflammatory cytokines and exacerbating metabolic stress at both systemic and tissue levels [25,39]. In line with the above, our results demonstrate that a hypercaloric diet induces a significant activation of NF-κB/p65 in all intestinal regions, reflecting the inflammatory state characteristic of obesity. However, we observed that BCP supplementation attenuates this activation in a regionally heterogeneous manner, while its effect was marked in the ileum; its modulatory capacity in the colon was substantially lower. This disparity suggests that, beyond the reported direct anti-inflammatory properties of BCP [24] there are colon-specific factors that limit its therapeutic efficacy.

The colon presents unique conditions that could explain this lower efficacy, a more abundant and diverse microbiota dominates its environment than in the small intestine, which generates a different metabolic environment with greater production of substances that could interfere with BCP [40]. In addition, in obesity, the colon develops more chronic and severe inflammation, creating a state of resistance where protective mechanisms such as the PPAR-γ pathway can become less sensitive [41].

This variability can be explained through obesity-induced intestinal dysbiosis, characterized by an imbalance in the Firmicutes/Bacteroidetes ratio [42,43] and a reduction in bacteria that produce protective metabolites, such as Akkermansia and Faecalibacterium [23]. This disruption not only compromises the integrity of the intestinal barrier by favoring endotoxin translocation and inflammation [44,45], but could also interfere with the pharmacodynamics of BCP. Previous studies support this hypothesis, demonstrating that BCP possesses selective antimicrobial activity and promotes the production of butyrate, an SCFA with anti-inflammatory and epithelial homeostasis-stabilizing effects [46,47]. However, in a colonic environment dominated by dysbiosis, BCP’s ability to restore a normal microbial profile could be compromised, thus limiting its impact on NF-κB/p65 and PPAR-γ signaling.

The regulation of the gut–brain axis depends on multiple signaling systems that integrate metabolic and neuroendocrine signals, critically influencing the development and maintenance of obesity. Within this axis, the cannabinoid receptors CNR1 and CNR2 participate in appetite modulation, energy homeostasis, and inflammatory response; while stimulation of CNR1 in the central nervous system (CNS) is associated with increased caloric intake, activation of CB2 shows anti-inflammatory and protective effects in various pathological models [21,24].

On the other hand, the FFAR3 receptor regulates the signaling of metabolites produced by the gut microbiota, modulating the secretion of intestinal hormones that impact satiety and metabolism [48]. The LEPR receptor is crucial for the inhibition of food intake and, in states of obesity, its resistance or desensitization contributes to hyperphagia [49]. Finally, the GHSR receptor stimulates the orexigenic signal, enhancing caloric intake. The balance between these receptors and their ligands is essential for gut–brain bidirectional communication, the mismatch of which has been directly associated with the pathophysiology of obesity [50].

Our results demonstrate that the HFD induces a significant decrease in FFAR3 expression, a finding consistent with previous studies that report alterations in sensitivity to SCFAs in states of obesity [27] . This reduction in FFAR3, due to the intestinal dysbiosis characteristic of obesity, compromises the bacterial production of SCFA and its ability to activate this receptor, thus affecting energy homeostasis and the secretion of intestinal hormones such as PYY and GLP-1 [48]. However, BCP supplementation showed a positive effect, increasing the expression of this gene in both the STD and HFD, suggesting an ability to modulate sensitivity to microbiota metabolite products. This finding aligns with evidence positioning BCP as a metabolic modulator capable of enhancing high-fat diet-induced dysfunction, likely through the regulation of G-protein-coupled receptor-dependent pathways [51].

The BCP’s mechanism of action on FFAR3 could involve multiple levels of regulation. By modulating the composition of the gut microbiota [23] BCP could support SCFA production, thereby restoring signaling through FFAR3. According to its anti-inflammatory activity, mediated in part by CNR2 activation [52] it could improve receptor sensitivity by reducing the inflammatory state characteristic of obesity. This hypothesis is reinforced by the well-known interaction between the endocannabinoid system and FFAR3 signaling, where both systems converge in regulating energy homeostasis [45,53,54].

Regarding the regulation of hunger and satiety, we observed that BCP modulates the expression of key receptors such as LEPR and GHSR. Increased LEPR expression suggests an improvement in leptin recognition [52], while a reduction in GHSR would indicate a decrease in orexigenic ghrelin signaling. These effects could be explained by BCP’s ability to reduce circulating leptin levels [23], thereby decreasing the leptin resistance state; and by modulating the activity of the endocannabinoid system, particularly through CB2 activation [24,46], which could improve ghrelin sensitivity.

In relation to receptors of the endocannabinoid system, the results of this study highlight the role of BCP as a relevant modulator, particularly on CNR2, for which BCP acts as an agonist [21,24,52].

According to Hashiesh et al. (2020) [52], BCP activates the CB2 receptor, which triggers several anti-inflammatory and immune-modulating responses. This activation translates into the inhibition of inflammatory mediators such as cytokines and prostaglandins, contributing to the reduction in inflammatory processes in various pathological models. On the other hand, Scandiffio et al. (2020) [46] explored the potential of BCP in the treatment of metabolic disorders. Although their study focused on the antioxidant and anti-inflammatory activity of BCP, they suggested that activation of the CB2 receptor could play a crucial role in improving insulin sensitivity and regulating energy metabolism, fundamental aspects in the management of obesity and its comorbidities. According to Youssef et al. (2019) [24], the effect of BCP was found in neuropathic pain models, and they observed that its action as a CB2 receptor agonist not only reduced the perception of pain, but also modulated the release of neurotransmitters related to inflammation and oxidative stress. These findings suggest that BCP, through activation of the CB2 receptor, may influence multiple physiological pathways, including those involved in energy homeostasis and appetite regulation.

In this study, the upward trend in CNR2 expression observed in the HFD + BCP group suggests that BCP could counteract the adverse effects of the hypercaloric diet on endocannabinoid signaling, a finding that aligns with previous research highlighting the role of this system in regulating appetite and energy metabolism [23,33]. Although no statistically significant differences were detected in the expression of CNR1, the observed trends indicate that BCP could be influencing energy homeostasis through multiple pathways, reinforcing its potential as an anorexigenic agent.

## 5. Conclusions

In conclusion, the present study, alongside the work of Rodríguez et al. (2022) [23], confirms the successful establishment of the diet-induced obesity model and replicates key findings on the metabolic benefits of BCP supplementation. The data reported by Rodríguez et al. [23] demonstrate a significant regulatory effect of BCP on body weight under metabolic challenge. In their study, metabolic data revealed significant differences between dietary regimens from week 3 (*p* < 0.001), with distinctions between hypercaloric groups (HFD vs. HFD + BCP) emerging by week 6 (*p* < 0.05) and persisting through week 16 (*p* < 0.01). Their results showed that the HFD group exhibited a significantly greater body weight compared to both the STD (*p* < 0.001) and STD + BCP (*p* < 0.0001) groups, while the trend toward reduced weight gain in the HFD + BCP group highlights BCP’s efficacy. This effect on body weight occurred despite their observation that animals on a standard diet consumed more food than those on a high-fat diet, suggesting that BCP improves metabolic efficiency rather than suppressing appetite. Furthermore, these immunometabolic improvements are consistent with previous research indicating that BCP acts as a nutraceutical agonist of CB2 and PPARγ receptors. This mechanism, as reported by Franco-Arroyo et al. (2022) [33], underpins its immunomodulatory effects, as evidenced by the modulation of key inflammatory mediators such as adiponectin, leptin, insulin, IL-6, TNF-α, and Toll-like receptor 4. Taken together, these findings and previous reports substantiate that BCP supplementation effectively ameliorates proinflammatory processes and improves overall metabolic homeostasis.

This study demonstrates that β-caryophyllene (BCP) exerts significant modulatory effects on key markers of the gut–brain axis in obese mice, evidenced by a tissue-specific attenuation of NF-κB/p65 activation and the concurrent modulation of integral communication systems, including the leptin/ghrelin axis, FFAR3, and endocannabinoid receptors. However, the observed regional variability and context-dependent efficacy suggest that BCP’s action is likely influenced by the local gut dysbiosis and metabolic state of the target tissue. Consequently, while BCP represents a promising multi-target therapeutic strategy, future research must elucidate its precise molecular mechanisms within the gut–brain axis and adopt a precision nutrition approach to tailor its application, necessitating rigorous clinical trials to validate its long-term efficacy and safety.

## Data Availability

The original contributions of this study are included in the article. Further information is available from the corresponding author upon reasonable request.

## Abbreviations

The following abbreviations are used in this manuscript:

BCA	Bicinchoninic Acid
BCP	β-Caryophyllene
CNR1	Cannabinoid Receptor 1
CNR2	Cannabinoid Receptor 2
CNS	Central Nervous System
FFAR3	Free Fatty Acid Receptor 3
GAPDH	Glyceraldehyde-3-Phosphate Dehydrogenase
GHSR	Growth Hormone Secretagogue Receptor (Ghrelin Receptor)
GLP-1	Glucagon-Like Peptide-1
HFD	High-Fat Diet
HFD + BCP	High-Fat Diet + β-Caryophyllene
IgG	Immunoglobulin G
IL-8	Interleukin 8
LEPR	Leptin Receptor
NF-κB/p65	Nuclear Factor Kappa B, p65 subunit
OD	Optical Density
PPARγ/PPAR-γ	Peroxisome Proliferator-Activated Receptor Gamma
PBS	Phosphate-Buffered Saline
PCR	Polymerase Chain Reaction
PVDF	Polyvinylidene Fluoride
qPCR	Quantitative Polymerase Chain Reaction
RIPA	Radioimmunoprecipitation Assay (lysis buffer)
RNA	Ribonucleic Acid
SCFA	Short-Chain Fatty Acids
SD	Standard Deviation
STD	Standard Diet
STD + BCP	Standard Diet + β-Caryophyllene
TNF-α	Tumor Necrosis Factor Alpha
TRIzol	Tri-Reagent for RNA isolation
WHO	World Health Organization

## Appendix A

### Appendix A.1. Calculation of the Sample Size

$$n=\frac{Z^{2}\sigma^{2}}{\delta^{2}}$$

In order to determine the number of mice per group to be used, the results obtained during the first pilot will be considered. The variable to consider will be the body weight of the mice on a hypercaloric diet. The calculation will be made based on the formula for estimating the mean:

*n* = sample size
Z = confidence level considering the p to be used = 1.96
σ = variation obtained in the measured parameter = 5.6
δ = desired variation in the parameter = 3

### Appendix A.2. Primers Used for the Amplification of the Genes of Interest

**Table A1:** Primers Employed in Gene Amplification Assays.

Forward Oligo	Sequence	Reverse Oligo	Sequence
FW1 -CNR1	CCTTGCAGATACCACCTTCC	REV1-CNR1	CTGAAGGAAGTTAGAGGGAATTTCTG
FW1-CNR2	CCTCGTACCTGTTCATCAGCAG	REV1-CNR2	GTGAAGGTCATGGTCACACTGC
FW1- FFAR3	GCTTCTTTCTTGGCAATTACTGG	REV1- FFAR3	GTTTAGCAAAAGTAAGTCCACAGC
FW1- GHSR	CTAACGTCACGCTGGACCTG	REV1- GHSR	GTTGCCCGAGATGCCCAC
FW1- LEPR	GCACTTAACCTGGCATATCCAATCTC	REV1- LEPR	GGATAACTCAGGAACGTAGATACCAC
FW1-GAPDH	GAAGGTCGGTGTGAACGGATTTGG	REV1-GAPDH	CGTGAGTGGAGTCATACTGGAACATG

## References

[1] Agus A., Clément K., Sokol H. (2021). Gut Microbiota-Derived Metabolites as Central Regulators in Metabolic Disorders. Gut.

[2] WHO Obesity. https://www.who.int/health-topics/obesity.

[3] Srivastava G., Apovian C.M. (2018). Current Pharmacotherapy for Obesity. Nat. Rev. Endocrinol..

[4] Gadde K.M., Apolzan J.W., Berthoud H.-R. (2018). Pharmacotherapy for Patients with Obesity. Clin. Chem..

[5] Kan H., Bae J.P., Dunn J.P., Buysman E.K., Gronroos N.N., Swindle J.P., Bengtson L.G.S., Ahmad N. (2023). Real-World Primary Nonadherence to Antiobesity Medications. J. Manag. Care Spec. Pharm..

[6] Rajan L., Palaniswamy D., Mohankumar S.K. (2020). Targeting Obesity with Plant-Derived Pancreatic Lipase Inhibitors: A Comprehensive Review. Pharmacol. Res..

[7] Sun W., Shahrajabian M.H., Cheng Q. (2021). Natural Dietary and Medicinal Plants with Anti-Obesity Therapeutics Activities for Treatment and Prevention of Obesity during Lock Down and in Post-COVID-19 Era. Appl. Sci..

[8] Zamani B., Daneshzad E., Siassi F., Guilani B., Bellissimo N., Azadbakht L. (2020). Association of Plant-Based Dietary Patterns with Psychological Profile and Obesity in Iranian Women. Clin. Nutr..

[9] Jupp P.W. (2020). Selected Environmental Factors in a Complex Systems Approach to Managing Obesity. Obes. Med..

[10] Karri S., Sharma S., Hatware K., Patil K. (2019). Natural Anti-Obesity Agents and Their Therapeutic Role in Management of Obesity: A Future Trend Perspective. Biomed. Pharmacother..

[11] Lu M., Cao Y., Xiao J., Song M., Ho C.-T. (2018). Molecular Mechanisms of the Anti-Obesity Effect of Bioactive Ingredients in Common Spices: A Review. Food Funct..

[12] Giuseppe D., Angela D., Davide R., Pamela M. (2017). Effects of a Combination of Berberis Aristata, Silybum Marianum and Monacolin on Lipid Profile in Subjects at Low Cardiovascular Risk; A Double-Blind, Randomized, Placebo-Controlled Trial. IJMS.

[13] Pirillo A., Catapano A.L. (2015). Berberine, a Plant Alkaloid with Lipid- and Glucose-Lowering Properties: From in Vitro Evidence to Clinical Studies. Atherosclerosis.

[14] Alway S.E., Bennett B.T., Wilson J.C., Edens N.K., Pereira S.L. (2014). Epigallocatechin-3-Gallate Improves Plantaris Muscle Recovery after Disuse in Aged Rats. Exp. Gerontol..

[15] Kim A.R., Kim K.M., Byun M.R., Hwang J.H., Park J.I., Oh H.T., Jeong M.G., Hwang E.S., Hong J.H. (2017). (-)-Epigallocatechin-3-Gallate Stimulates Myogenic Differentiation through TAZ Activation. Biochem. Biophys. Res. Commun..

[16] Mirza K.A., Pereira S.L., Edens N.K., Tisdale M.J. (2014). Attenuation of Muscle Wasting in Murine C2C12 Myotubes by Epigallocatechin-3-Gallate. J. Cachexia Sarcopenia Muscle.

[17] Adibian M., Hodaei H., Nikpayam O., Sohrab G., Hekmatdoost A., Hedayati M. (2019). The Effects of Curcumin Supplementation on High-sensitivity C-reactive Protein, Serum Adiponectin, and Lipid Profile in Patients with Type 2 Diabetes: A Randomized, Double-blind, Placebo-controlled Trial. Phytother. Res..

[18] Chong P.-W., Beah Z.-M., Grube B., Riede L. (2014). IQP-GC-101 Reduces Body Weight and Body Fat Mass: A Randomized, Double-Blind, Placebo-Controlled Study. Phytother. Res..

[19] Gonnelli S., Caffarelli C., Stolakis K., Cuda C., Giordano N., Nuti R. (2015). Efficacy and Tolerability of a Nutraceutical Combination (Red Yeast Rice, Policosanols, and Berberine) in Patients with Low-Moderate Risk Hypercholesterolemia: A Double-Blind, Placebo-Controlled Study. Curr. Ther. Res..

[20] Widjajakusuma E.C., Jonosewojo A., Hendriati L., Wijaya S., Ferawati, Surjadhana A., Sastrowardoyo W., Monita N., Muna N.M., Fajarwati R.P. (2019). Phytochemical Screening and Preliminary Clinical Trials of the Aqueous Extract Mixture of Andrographis Paniculata (Burm. f.) Wall. Ex Nees and Syzygium Polyanthum (Wight.) Walp Leaves in Metformin Treated Patients with Type 2 Diabetes. Phytomedicine.

[21] Hashiesh H.M., Azimullah S., Meeran M.F.N., Saraswathiamma D., Jha N.K., Sadek B., Adeghate E., Tariq S., Marzooqi S.A., Ojha S. (2023). β-Caryophyllene, a Dietary Phytocannabinoid, Alleviates Diabetic Cardiomyopathy in Mice by Inhibiting Oxidative Stress and Inflammation Activating Cannabinoid Type-2 Receptors. ACS Pharmacol. Transl. Sci..

[22] Alizadeh S., Djafarian K., Mofidi Nejad M., Yekaninejad M.S., Javanbakht M.H. (2022). The Effect of β-Caryophyllene on Food Addiction and Its Related Behaviors: A Randomized, Double-Blind, Placebo-Controlled Trial. Appetite.

[23] Rodríguez-Mejía U.U., Viveros-Paredes J.M., Zepeda-Morales A.S.M., Carrera-Quintanar L., Zepeda-Nuño J.S., Velázquez-Juárez G., Delgado-Rizo V., García-Iglesias T., Camacho-Padilla L.G., Varela-Navarro E. (2022). β-Caryophyllene: A Therapeutic Alternative for Intestinal Barrier Dysfunction Caused by Obesity. Molecules.

[24] Youssef D.A., El-Fayoumi H.M., Mahmoud M.F. (2019). Beta-Caryophyllene Protects against Diet-Induced Dyslipidemia and Vascular Inflammation in Rats: Involvement of CB2 and PPAR-γ Receptors. Chem. Biol. Interact..

[25] Dammann K., Khare V., Lang M., Claudel T., Harpain F., Granofszky N., Evstatiev R., Williams J.M., Pritchard D.M., Watson A. (2015). PAK1 Modulates a PPARγ/NF-κB Cascade in Intestinal Inflammation. Biochim. Biophys. Acta (BBA) Mol. Cell Res..

[26] Hong F., Pan S., Guo Y., Xu P., Zhai Y. (2019). PPARs as Nuclear Receptors for Nutrient and Energy Metabolism. Molecules.

[27] Kasubuchi M., Hasegawa S., Hiramatsu T., Ichimura A., Kimura I. (2015). Dietary Gut Microbial Metabolites, Short-chain Fatty Acids, and Host Metabolic Regulation. Nutrients.

[28] Cryan J.F., O’Riordan K.J., Cowan C.S.M., Sandhu K.V., Bastiaanssen T.F.S., Boehme M., Codagnone M.G., Cussotto S., Fulling C., Golubeva A.V. (2019). The Microbiota-Gut-Brain Axis. Physiol. Rev..

[29] O’Riordan K.J., Moloney G.M., Keane L., Clarke G., Cryan J.F. (2025). The Gut Microbiota-Immune-Brain Axis: Therapeutic Implications. CR Med..

[30] Motawi T.K., Shaker O.G., Ismail M.F., Sayed N.H. (2017). Peroxisome Proliferator-Activated Receptor Gamma in Obesity and Colorectal Cancer: The Role of Epigenetics. Sci. Rep..

[31] Krentz A.J., Bailey C.J. (2005). Oral Antidiabetic Agents: Current Role in Type 2 Diabetes Mellitus. Drugs.

[32] Wang L., Xu H., Tan B., Qu Y., Liu H., Deng H., Chen Y., Wang R., Tian J., Zhu J. (2022). Gut Microbiota and Its Derived SCFAs Regulate the HPGA to Reverse Obesity-Induced Precocious Puberty in Female Rats. Front. Endocrinol..

[33] Franco-Arroyo N.N., Viveros-Paredes J.M., Zepeda-Morales A.S.M., Roldán E., Márquez-Aguirre A.L., Zepeda-Nuño J.S., Velázquez-Juárez G., Fafutis-Morris M., López-Roa R.I. (2022). β-Caryophyllene, a Dietary Cannabinoid, Protects Against Metabolic and Immune Dysregulation in a Diet-Induced Obesity Mouse Model. J. Med. Food.

[34] Wang S., Lin Y., Gao L., Yang Z., Lin J., Ren S., Li F., Chen J., Wang Z., Dong Z. (2022). PPAR-γ Integrates Obesity and Adipocyte Clock through Epigenetic Regulation of Bmal1. Theranostics.

[35] Dubuquoy L., Rousseaux C., Thuru X., Peyrin-Biroulet L., Romano O., Chavatte P., Chamaillard M., Desreumaux P. (2006). PPARgamma as a New Therapeutic Target in Inflammatory Bowel Diseases. Gut.

[36] Su W., Bush C.R., Necela B.M., Calcagno S.R., Murray N.R., Fields A.P., Thompson E.A. (2007). Differential Expression, Distribution, and Function of PPAR-Gamma in the Proximal and Distal Colon. Physiol. Genom..

[37] Engin A., Engin A.B., Engin A. (2024). Lipid Storage, Lipolysis, and Lipotoxicity in Obesity. Obesity and Lipotoxicity.

[38] Roberts L.D., Murray A.J., Menassa D., Ashmore T., Nicholls A.W., Griffin J.L. (2011). The Contrasting Roles of PPARδ and PPARγ in Regulating the Metabolic Switch between Oxidation and Storage of Fats in White Adipose Tissue. Genome Biol..

[39] Serra M.P., Serra M.P., Boi M., Boi M., Carta A., Carta A., Murru E., Murru E., Carta G., Carta G. (2022). Anti-Inflammatory Effect of Beta-Caryophyllene Mediated by the Involvement of TRPV1, BDNF and trkB in the Rat Cerebral Cortex after Hypoperfusion/Reperfusion. Int. J. Mol. Sci..

[40] Zhao J., Zhao R., Cheng L., Yang J., Zhu L. (2018). Peroxisome Proliferator-Activated Receptor Gamma Activation Promotes Intestinal Barrier Function by Improving Mucus and Tight Junctions in a Mouse Colitis Model. Dig. Liver Dis..

[41] Caioni G., Viscido A., d’Angelo M., Panella G., Castelli V., Merola C., Frieri G., Latella G., Cimini A., Benedetti E. (2021). Inflammatory Bowel Disease: New Insights into the Interplay between Environmental Factors and PPARγ. Int. J. Mol. Sci..

[42] Hildebrandt M.A., Hoffmann C., Sherrill–Mix S.A., Keilbaugh S.A., Hamady M., Chen Y.-Y., Knight R., Ahima R.S., Bushman F., Wu G.D. (2009). High-Fat Diet Determines the Composition of the Murine Gut Microbiome Independently of Obesity. Gastroenterology.

[43] Ley R.E., Bäckhed F., Turnbaugh P., Lozupone C.A., Knight R.D., Gordon J.I. (2005). Obesity Alters Gut Microbial Ecology. Proc. Natl. Acad. Sci. USA.

[44] Cani P.D., Plovier H., Van Hul M., Geurts L., Delzenne N.M., Druart C., Everard A. (2016). Endocannabinoids—At the Crossroads between the Gut Microbiota and Host Metabolism. Nat. Rev. Endocrinol..

[45] Muccioli G.G., Naslain D., Bäckhed F., Reigstad C.S., Lambert D.M., Delzenne N.M., Cani P.D. (2010). The Endocannabinoid System Links Gut Microbiota to Adipogenesis. Mol. Syst. Biol..

[46] Scandiffio R., Geddo F., Cottone E., Querio G., Antoniotti S., Gallo M.P., Maffei M.E., Bovolin P. (2020). Protective Effects of (E)-β-Caryophyllene (BCP) in Chronic Inflammation. Nutrients.

[47] Yoo H.-J., Jwa S.-K. (2019). Efficacy of β-Caryophyllene for Periodontal Disease Related Factors. Arch. Oral Biol..

[48] Kimura I., Ichimura A., Ohue-Kitano R., Igarashi M. (2020). Free Fatty Acid Receptors in Health and Disease. Physiol. Rev..

[49] O’Rahilly S., Farooqi I.S., Loscalzo J., Fauci A., Kasper D., Hauser S., Longo D., Jameson J.L. (2022). Biopatología de La Obesidad. Harrison. Principios de Medicina Interna.

[50] Włodarczyk M., Nowicka G. (2019). Obesity, DNA Damage, and Development of Obesity-Related Diseases. Int. J. Mol. Sci..

[51] Cook T.M., Gavini C.K., Jesse J., Aubert G., Gornick E., Bonomo R., Gautron L., Layden B.T., Mansuy-Aubert V. (2021). Vagal Neuron Expression of the Microbiota-Derived Metabolite Receptor, Free Fatty Acid Receptor (FFAR3), Is Necessary for Normal Feeding Behavior. Mol. Metab..

[52] Hashiesh H.M., Meeran M.F.N., Sharma C., Sadek B., Kaabi J.A., Ojha S.K. (2020). Therapeutic Potential of β-Caryophyllene: A Dietary Cannabinoid in Diabetes and Associated Complications. Nutrients.

[53] De Vadder F., Kovatcheva-Datchary P., Goncalves D., Vinera J., Zitoun C., Duchampt A., Bäckhed F., Mithieux G. (2014). Microbiota-Generated Metabolites Promote Metabolic Benefits via Gut-Brain Neural Circuits. Cell.

[54] Kimura I., Inoue D., Maeda T., Hara T., Ichimura A., Miyauchi S., Kobayashi M., Hirasawa A., Tsujimoto G. (2011). Short-Chain Fatty Acids and Ketones Directly Regulate Sympathetic Nervous System via G Protein-Coupled Receptor 41 (GPR41). Proc. Natl. Acad. Sci. USA.

